# Family History and Adherence to Ideal Cardiovascular Health: Counteracting Impacts on the Onset Age of Type 2 Diabetes and Subsequent Cardiovascular Disease Risk

**DOI:** 10.1111/1753-0407.70256

**Published:** 2026-08-05

**Authors:** Lihong Zhang, Jinyue Li, Jianxin Li, Wanyue Wang, Shufeng Chen, Jie Cao, Keyong Huang, Jichun Chen, Xiaoqing Liu, Xueli Yang, Chong Shen, Ling Yu, Yingxin Zhao, Xianping Wu, Liancheng Zhao, Ying Li, Dongsheng Hu, Jianfeng Huang, Dongfeng Gu, Xiangfeng Lu, Fangchao Liu

**Affiliations:** ^1^ Endocrinology Centre Fuwai Hospital, National Center for Cardiovascular Diseases, Chinese Academy of Medical Sciences and Peking Union Medical College Beijing China; ^2^ Department of Epidemiology, State Key Laboratory of Cardiovascular Disease Fuwai Hospital, National Center for Cardiovascular Diseases, Chinese Academy of Medical Sciences and Peking Union Medical College Beijing China; ^3^ Division of Epidemiology Guangdong Provincial People's Hospital and Cardiovascular Institute Guangzhou China; ^4^ Tianjin Key Laboratory of Environment, Nutrition and Public Health; Department of Occupational and Environmental Health, School of Public Health Tianjin Medical University Tianjin China; ^5^ Department of Epidemiology and Biostatistics School of Public Health, Nanjing Medical University Nanjing China; ^6^ Department of Cardiology Shengli Clinical Medical College of Fujian Medical University, Fujian Provincial Hospital, Fuzhou University Affiliated Provincial Hospital Fuzhou China; ^7^ Cardio‐Cerebrovascular Control and Research Center Institute of Basic Medicine, Shandong Academy of Medical Sciences Jinan China; ^8^ Center for Chronic and Noncommunicable Disease Control and Prevention Sichuan Center for Disease Control and Prevention Chengdu China; ^9^ Department of Epidemiology and Health Statistics, College of Public Health Zhengzhou University Zhengzhou China; ^10^ Department of Biostatistics and Epidemiology School of Public Health, Shenzhen University Health Science Center Shenzhen China; ^11^ School of Public Health and Emergency Management School of Medicine, Southern University of Science and Technology Shenzhen China

**Keywords:** cardiovascular disease, cardiovascular health, family history, onset age, type 2 diabetes

## Abstract

**Background:**

To analyze the interplay between family history of type 2 diabetes (T2D) and cardiovascular health (CVH) in relation to T2D onset age and subsequent cardiovascular disease (CVD) risk.

**Methods:**

A total of 79 831 participants were included to investigate the association between family history and T2D onset age. Then, 7387 diagnosed T2D patients were 1:1 matched with non‐T2D individuals to analyze the association of T2D onset age with subsequent CVD risk. The benefit of good CVH was further assessed. Stratified Cox regression and conditional Cox regression were performed to estimate hazard ratios (HRs) and 95% confidence intervals (CIs).

**Results:**

Family history was associated with younger T2D onset, with an approximately 2.4‐year earlier onset of T2D, which further increased the subsequent risk of CVD. Compared with individuals without family history, those with family history had HRs (95% CIs) of 3.911 (3.041, 5.032), 3.785 (3.385, 4.233), 3.627 (3.340, 3.938), 3.465 (3.189, 3.764), and 3.169 (2.787, 3.603) for T2D onset at < 40, 40–50, 50–60, 60–70, and ≥ 70 years old, respectively (*P*_interaction_ = 0.007). Among individuals with family history, HR (95% CI) for incident CVD was 3.598 (1.372, 9.433) for those diagnosed T2D < 50 years compared with those without T2D, while the HR (95% CI) was 2.336 (1.232, 4.428) among those without family history. However, high CVH could mitigate risk of young‐onset T2D, and could further decrease CVD risk after their T2D diagnosis.

**Conclusions:**

Having family history of T2D increased susceptibility to young‐onset T2D and subsequent CVD risks, while ideal CVH could counteract these risks, highlighting the necessity of early screening and intervention.

## Highlights

- The influence of family history on young‐onset type 2 diabetes (T2D) and subsequent cardiovascular disease (CVD) risk has not been well quantified, and the extent to which ideal cardiovascular health (CVH) offsets these risks is unclear.
- A prospective cohort of Chinese adults revealed that family history increased young‐onset T2D risk and exacerbated subsequent CVD risk in individuals diagnosed with T2D early.
- Ideal CVH not only delayed T2D onset associated with family history, but also attenuated CVD risk attributed to young‐onset T2D.

## Graphical abstract

Family history serves as a crucial indicator for identifying high‐risk populations for early‐onset T2D, highlighting the importance of regular screening and early CVH interventions.

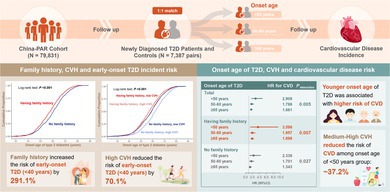

## 1 Introduction

With the development of socioeconomics and changes in lifestyle, there is a concerning trend of diabetes manifesting at younger ages. Data from the International Diabetes Federation's Diabetes Atlas show that the global prevalence of diabetes among individuals aged 20–39 increased from 2.9% in 2013 to 3.8% in 2021, with further increases expected [1, 2]. Since early‐onset type 2 diabetes (T2D) (usually defined as diagnosis at < 40 years of age) is associated with more rapid deterioration in β‐cell function, insulin resistance, and longer disease burden, there is an increased risk of adverse cardiovascular events and reduced life expectancy, imposing a significant burden on individuals and society [3, 4, 5]. Thus, identifying risk factors of early‐onset T2D has important public health implications for early prevention and control.

Evidence increasingly shows that family history is a significant risk factor for T2D, encompassing shared genetic predispositions, family behaviors, socioeconomic status, and environmental exposures [6]. However, studies investigating the association between family history and T2D onset age remain insufficient. Existing studies used cross‐sectional or small retrospective cohort designs, limiting accurate assessment of young‐onset T2D risk associated with family history and lacking robust evidence from large‐scale prospective cohort studies [7, 8, 9]. Quantitative assessment of the influence of family history on young‐onset T2D can facilitate early identification of high‐risk groups and enable early prevention of T2D. Moreover, several studies have suggested that family history of T2D was also associated with microvascular and macrovascular complications [10, 11]. Given that individuals diagnosed with T2D early often experience higher cardiovascular disease (CVD) incidence, mortality, and reduced life expectancy, the combined impact of family history and young‐onset T2D warrants greater attention [12].

However, family history is a nonmodifiable risk factor, highlighting the importance of addressing modifiable risk factors to empower individuals in their health management [13]. Currently, there exists a significant research gap regarding effective prevention and intervention for young adults with T2D, as existing guidelines lack specific CVD risk reduction recommendations for this demographic, presenting challenges for self‐management [2, 14, 15]. The American Heart Association (AHA) introduced new cardiovascular health (CVH) metrics, comprising lifestyle and metabolic factors, which are closely associated with reduced risk of both T2D and CVD [16, 17, 18]. Exploring whether adherence to optimal CVH can delay the onset of T2D among individuals with a family history of T2D and reduced CVD risk may support early detection and intervention strategies, thereby reducing complications and the socioeconomic burden.

In this study, we used data from a prospective, population‐based cohort to investigate two study objectives: (1) To analyze the association between family history of T2D and T2D onset age, as well as the protective impact of ideal CVH and (2) to examine the association between T2D onset age and CVD incidence according to family history, and explore whether ideal CVH could reduce CVD risk across different onset age groups. The research framework was illustrated in Figure S1.

## 2 Methods

### 2.1 Study Population

The Prediction for Atherosclerotic CVD Risk in China (China‐PAR) project is a large‐scale cohort that includes participants aged 18–74 in 15 provinces and cities nationwide. Details on study design, methodology, and participant characteristics have been published previously [19]. This study draws from three subcohorts, including the China Multi‐Center Collaborative Study of Cardiovascular Epidemiology (ChinaMUCA 1998), the International Collaborative Study of CVD in Asia (InterASIA), and the Community Intervention of Metabolic Syndrome in China and Chinese Family Health Study (CIMIC). Briefly, baseline investigations of ChinaMUCA 1998 and InterASIA were conducted in 1998 and 2000–2001, respectively, followed by follow‐up investigations in 2007–2008 and 2012–2015. CIMIC had its baseline investigation in 2007–2008 and followed up in 2012–2015. The study was approved by the Institutional Review Board at Fuwai Hospital in Beijing, and followed the Declaration of Helsinki, with written informed consent obtained from all participants.

Following the current study design, we first excluded 8185 subjects out of the initial 113 448 who were lost to follow‐up, followed by the exclusion of 44 subjects lacking the onset age of T2D, 1932 subjects with T2D at baseline, and 23 456 subjects missing fasting blood glucose (FBG) levels at either baseline or follow‐up. A total of 79 831 people were included in the analysis regarding the association of family history and CVH with onset age of T2D. For the subsequent analysis of CVD outcomes, we further excluded 1324 individuals who had developed CVD at baseline or before the onset of T2D, and then matched new‐onset T2D patients with participants without T2D during the entire follow‐up period (until 2015) by age (±1 year), sex (male/female), follow‐up time (±1 year), living region (northern/southern China), subcohort (ChinaMUCA 1998/InterASIA/CIMIC) and family history of T2D (yes/no) at a ratio of 1:1. Finally, we obtained a total of 7387 matched pairs of new‐onset T2D cases and controls, respectively. The flow diagram of inclusion and exclusion was shown in Figure S2.

### 2.2 Data Collection and Definition of Family History and CVH

At baseline and follow‐up visits, standardized questionnaires were used to collect demographic characteristics, family history, disease history, and lifestyle factors through face‐to‐face interviews. Clinical examinations including height, weight, and BP measurements were performed following standardized protocol. Participants provided fasting blood samples for biochemical tests.

Family history of T2D was collected via questionnaire. Individuals reporting a parent or sibling with T2D at either baseline or follow‐up were considered to have a family history of T2D; otherwise, they were considered to have no family history of T2D.

CVH was defined according to the AHA Life's Essential 8 [16], including four health behaviors (dietary behavior, physical activity, smoking, and sleep duration) and four health metabolic factors (body mass index [BMI], blood pressure [BP], non‐high‐density lipoprotein cholesterol [non‐HDLC], and blood glucose). Details on the definition of each ideal CVH factor were shown in Table S1. In particular, a modified dietary behavior score was defined based on the AHA's recommendations and the dietary guidelines for Chinese residents [20, 21]. The individual CVH metric was scored from 0 to 100 points. When analyzing the influence of CVH on the onset age of T2D, the overall CVH score was calculated as the unweighted average of seven metric scores (excluding blood glucose levels), ranging from 0 to 100 points, which was then classified as low, intermediate, and high CVH groups according to < 50, 50–80, and ≥ 80 points. When analyzing the influence of CVH on the CVD risk, the overall CVH score was calculated as the unweighted average of all eight metrics scores, and its range was also from 0 to 100 points. To obtain higher statistical power, we merged the intermediate (50–80 points) and high (≥ 80 points) CVH categories into medium‐high CVH category (≥ 50 points) for comparison against the low CVH group.

### 2.3 Covariates Assessment

Potential covariates in this study included sex, living region, urban area, education level, current smoking status, alcohol drinking status, physical activity, BMI, hypertension, dyslipidemia, family history of CVD, and subcohorts. Living region was categorized into northern and southern China. Education level was divided into below high school and high school or above. Current smokers were those who smoked at least 20 packs (or 500 g of dry tobacco) during their lifetime or consumed at least one cigarette per day for 1 year or more, and were not currently quitting. Physical activity was dichotomized according to whether they met the recommended criteria (moderate‐intensity physical activity > 150 min/week or vigorous‐intensity physical activity > 75 min/week, or equivalent amounts of these two strengths). BMI was calculated by dividing weight (kg) by the square of height (m^2^). BP was measured using a standardized mercury sphygmomanometer or an electronic sphygmomanometer in a quiet room after subjects had been seated for 5 min. Trained staff obtained three readings at 30‐s intervals, and the average value was used in the analysis. After the collection of fasting blood samples, total cholesterol (TC) and HDLC levels were assessed by the oxidase method and direct chemically modified enzyme method, respectively. Family history of CVD was defined as a parent or sibling diagnosed with CVD. Covariates adjusted in the model for the association between family history and T2D onset age were evaluated at baseline, and those for the association between T2D onset age and CVD risk were the values from the most recent measurement after the onset of T2D.

### 2.4 Outcome Determination

The outcomes were the T2D onset at different ages and incident CVD. T2D was defined as FBG ≥ 126 mg/dL or the use of any oral hypoglycemic agent or insulin [22]. To determine the date of T2D incidence, we used the earliest available date among the following sources: (1) the self‐reported date of first clinical diagnosis; (2) the initiation date of hypoglycemic medication as recorded in medical records; or (3) the date of the blood sampling that first met the FBG diagnostic criterion. This hierarchical approach was designed to capture the earliest indicator of disease onset. T2D onset age was calculated by subtracting the participant's date of birth from this determined onset date. Incident CVD was defined according to the International Classification of Diseases, 10th edition (ICD‐10, I00–I99), with a composition of nonfatal acute myocardial infarction, unstable angina, heart failure, stroke, and cardiovascular death events. Data on outcomes were collected through face‐to‐face interviews, hospital records, and death certificates, and finally reviewed and confirmed by the endpoint expert committee at Fuwai Hospital.

### 2.5 Statistical Analysis

Characteristics were described as mean ± standard deviation (SD) for continuous variables, and number (percentage) for categorical variables. Group differences were compared using *t*‐test or ANOVA for continuous variables and the chi‐square test for categorical variables. We first performed Kaplan–Meier analysis with the log‐rank test to compare cumulative T2D incidence between those with or without family history of T2D. Then, age was categorized into five time‐varying intervals (< 40, 40–50, 50–60, 60–70, and ≥ 70 years), and individuals were allowed to enter multiple higher age groups as time went by, until T2D onset, death, loss to follow‐up, or the end of the last investigation [23]. Stratified Cox proportional hazards regression was used to estimate the hazard ratios (HRs) and 95% confidence intervals (95% CIs) for T2D risk associated with family history among the five age groups, with age as the time scale. The interaction effect was evaluated by likelihood ratio test after incorporating the product of onset age group and family history in the model. Additionally, we further estimated the differences in onset age of T2D between the groups with or without family history by restricted mean survival time (RMST) models [24]. To analyze the associations of T2D onset age on CVD incidence, considering the sample size of each group, individuals were divided into three groups based on T2D onset age: < 50, 50–60, and ≥ 60 years. After random matching, we subsequently conducted conditional Cox proportional hazards regression to estimate the HR (95% CI) for CVD incidence comparing new‐onset T2D patients with controls in each age group, stratified by family history [25]. Likelihood ratio test was also used to evaluate the interaction effect, incorporating the product of onset age group and T2D in the model.

Furthermore, to explore the effects of CVH on young‐onset T2D and subsequent CVD risk, we first divided patients into four groups: (1) family history with low CVH, (2) family history with high CVH, (3) no family history with low CVH, and (4) no family history with high CVH, comparing their Kaplan–Meier curves. We also assessed the association between CVH and T2D risk across different onset ages and family history groups, and investigated the associations between CVH and CVD risk across each T2D onset age group. The role of individual CVH components was also analyzed. We conducted subgroup analyses by sex to explore sex differences. In the sensitivity analysis, we employed the Fine‐Gray competing‐risk model to account for competing risk. All analyses were conducted using SAS version 9.4 and R software version 4.2.0. Statistical tests were two‐sided, and *p* < 0.05 was considered statistically significant.

## 3 Results

### 3.1 Study Characteristics

Among the 79 831 participants included in the analysis, 12 028 individuals had a family history of T2D, accounting for 15.1%. Baseline characteristics of the participants categorized by family history status are shown in Table S2. In total, the average age of participants was 51.5 ± 11.7 years, with 39.7% being male. Compared with individuals without family history, those with family history had lower CVH scores, and higher prevalence of overweight as well as higher levels of BP, FBG, TC, and LDLC.

Among the included T2D patients and matched controls, patients with new‐onset T2D had a higher prevalence of overweight and higher levels of BP, TC, and LDLC, and lower CVH score, compared with control subjects. Other characteristics are shown in Table S3.

### 3.2 Associations of Family History With T2D Risk Among Different Onset Age Groups

**FIGURE 1:**
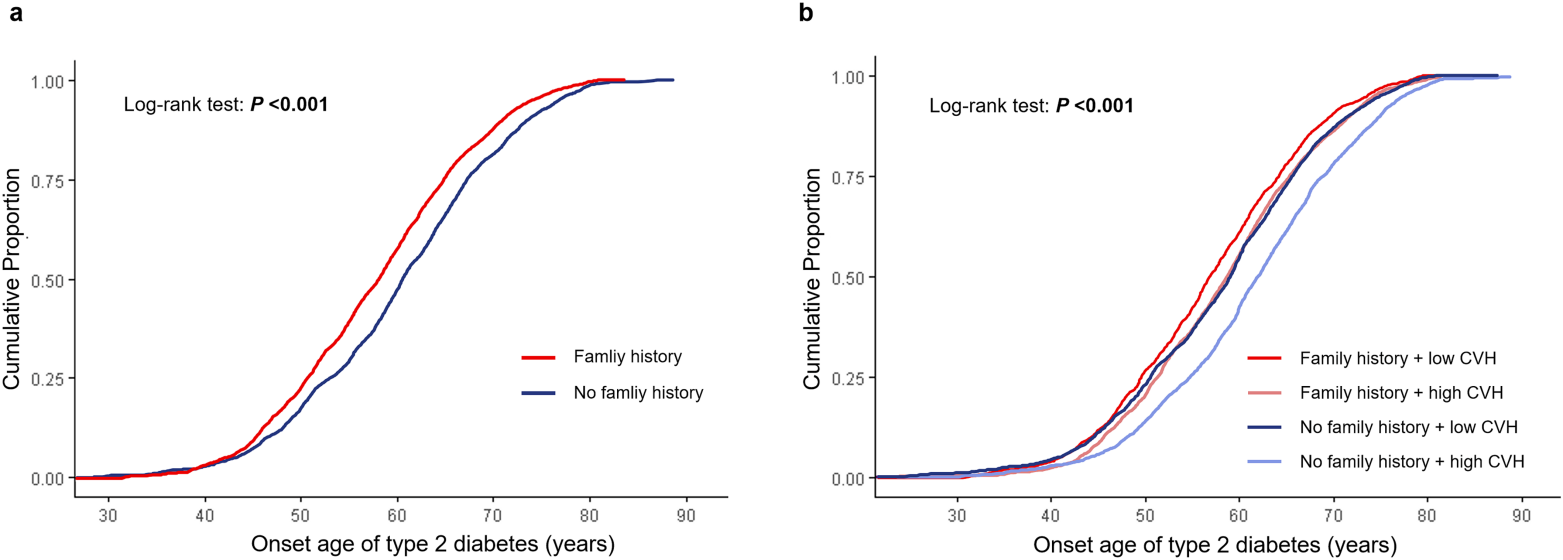
Cumulative incidence rate of T2D stratified by (a) family history and (b) family history combined with CVH score group. CVH, cardiovascular health; T2D, type 2 diabetes.

During a mean follow‐up period of 7.2 years, a total of 7387 incident cases of T2D were identified. The cumulative proportion along with the onset age of T2D demonstrated a significantly earlier onset of T2D among individuals with family history (*p* < 0.001) (Figure 1a). The mean age at T2D onset was 57.8 ± 12.1 years for those with family history, compared to 60.3 ± 12.0 years for those without family history (*p* < 0.001). Moreover, the incidence rate of T2D was substantially higher among individuals with family history than those without, and this incidence rate increased across older age groups with the increase of onset age. The results of the RMST model indicated that the onset age of T2D in individuals with family history was approximately 2.4 years earlier than that in those without family history (Figure S3). Across all onset age groups, T2D risk was significantly elevated among individuals with family history, with the relative risk increasing as the onset age decreased. In the fully adjusted models, compared to individuals without family history, those with family history had HRs (95% CIs) of 3.911 (3.041, 5.032), 3.785 (3.385, 4.233), 3.627 (3.340, 3.938), 3.465 (3.189, 3.764), and 3.169 (2.787, 3.603) for T2D onset at < 40, 40–50, 50–60, 60–70, and ≥ 70 years old, respectively. A significant multiplicative interaction was observed between family history and age groups (*P*_interaction_ = 0.007) (Table 1). Subgroup analyses revealed that women with family history had elevated risk of young‐onset T2D, with corresponding HRs (95% CIs) of 4.175 (2.951, 5.906), 3.697 (3.198, 4.274), 3.542 (3.188, 3.936), 3.553 (3.195, 3.950), and 2.898 (2.458, 3.416) across the respective onset age groups compared to those without family history (*P*_interaction_ = 0.002), while this trend was less pronounced in men (*P*_interaction_ = 0.076) (Table S4).

**TABLE 1:** The association of family history with the incidence risk of T2D by onset age of T2D.

Onset age groups	T2D cases/participants, *N*	IR, per 1000 person‐years	Model 1, HR (95% CI)	Model 2, HR (95% CI)	Model 3, HR (95% CI)
< 40 years					
No family history	157/12124	3.50	1.000 (Ref.)	1.000 (Ref.)	1.000 (Ref.)
Family history	112/2200	15.26	4.215 (3.303, 5.379)	4.216 (3.298, 5.388)	3.911 (3.041, 5.032)
40–50 years					
No family history	733/25455	5.94	1.000 (Ref.)	1.000 (Ref.)	1.000 (Ref.)
Family history	615/5310	23.53	3.945 (3.344, 4.391)	4.156 (3.727, 4.635)	3.785 (3.385, 4.233)
50–60 years					
No family history	1490/34527	9.77	1.000 (Ref.)	1.000 (Ref.)	1.000 (Ref.)
Family history	1034/6488	36.96	3.814 (3.523, 4.129)	3.961 (3.654, 4.294)	3.627 (3.340, 3.938)
60–70 years					
No family history	1750/28659	14.63	1.000 (Ref.)	1.000 (Ref.)	1.000 (Ref.)
Family history	936/4368	55.65	3.818 (3.527, 4.134)	3.822 (3.526, 4.142)	3.465 (3.189, 3.764)
≥ 70 years					
No family history	946/12494	17.97	1.000 (Ref.)	1.000 (Ref.)	1.000 (Ref.)
Family history	377/1595	62.56	3.561 (3.159, 4.014)	3.614 (3.396, 4.086)	3.169 (2.787, 3.603)
*P*_interaction_			0.180	0.039	0.007

*Note:* Model 1 was crude model; Model 2 was adjusted for sex, living region, and urban area; Model 3 was adjusted for sex, living region, urban area, education level, current smoking status, alcohol drinking status, physical activity, BMI, hypertension, dyslipidemia, and subcohorts. *P*_interaction_ represented the significance of interaction effect between family history and age groups.

Abbreviations: 95% CI, 95% confidence interval; BMI, body mass index; HR, hazard ratio; IR, incidence rate; T2D, type 2 diabetes.

### 3.3 Association of Onset Age of T2D With Risk of CVD Incident Risk Stratified by Family History

**FIGURE 2:**
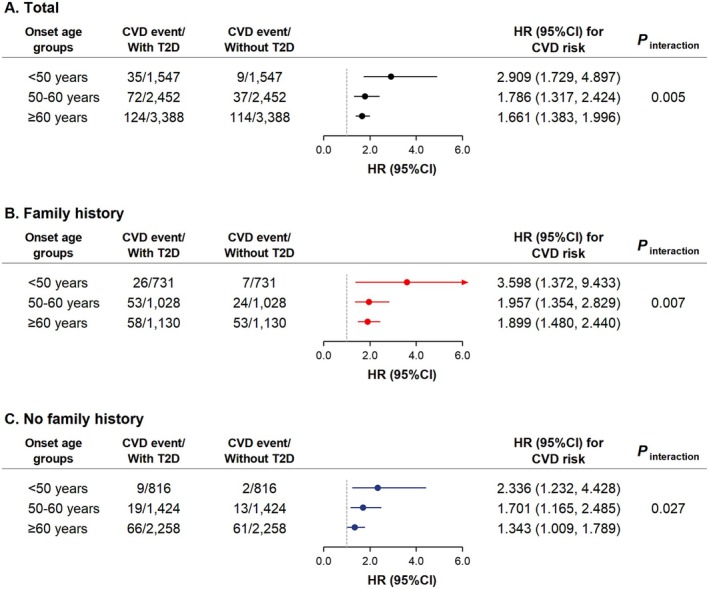
The association of T2D with different onset age with CVD risk among total population (A), individuals with (B) and without (C) family history. 95% CI, 95% confidence interval; BMI, body mass index; CVD, cardiovascular disease; HR, hazard ratio; T2D, type 2 diabetes. HRs (95% CIs) for CVD risk associated with T2D across three age groups were calculated using Cox regression models, with the population without T2D serving as the reference group. Models were adjusted for urban area, education level, current smoking status, alcohol drinking status, physical activity, family history of CVD, BMI, hypertension, dyslipidemia, and hypoglycemic medication use. *P*_interaction_ represented the significance of the interaction effect between onset age and T2D.

The association between the onset age of T2D and the risk of incident CVD was displayed in Figure 2, suggesting that the younger the age of T2D onset, the higher the risk of CVD incidence (*P*_interaction_ = 0.005). In multivariable‐adjusted models, individuals with T2D onset at age < 50 years, 50–60 years, and ≥ 60 years had HRs (95% CIs) of 2.909 (1.729, 4.897), 1.786 (1.317, 2.424), and 1.661 (1.383, 1.996) for incident CVD, respectively, compared with individuals without T2D. Furthermore, we found that the association between an earlier age of T2D and the risk of CVD was stronger in individuals with family history. Specifically, in the group with family history, the HRs (95% CIs) for CVD incidence in T2D patients with onset age of < 50, 50–60, and ≥ 60 years were 3.598 (1.372, 9.433), 1.957 (1.354, 2.829), and 1.899 (1.480, 2.440), whereas the corresponding HRs (95% CIs) were 2.336 (1.232, 4.428), 1.701 (1.165, 2.485), and 1.343 (1.009, 1.789) in the group without family history, respectively. The onset age group and T2D had significant multiplicative interactions in the subgroup with and without family history (*P*_interaction_ = 0.007 and 0.027). The trend of the association between T2D and CVD across different onset age groups and the interaction between onset age group and T2D were observed in both men and women (*P*_interaction_ = 0.029 and 0.003) (Figure S4).

### 3.4 Influence of CVH on the Risk of T2D and Related CVD Risk Among Different Onset Age Groups

The Kaplan–Meier curves showed that high CVH might moderately delay the onset of T2D in both participants with and without family history, and that the cumulative proportion defined by onset age in individuals with family history and high CVH was similar to that in those without family history but low CVH (Figure 1b).

Across all age groups, the risk of T2D progressively decreased with increasing CVH level, and this protective impact became more pronounced with younger T2D onset ages (Table 2). Results from the fully adjusted model showed that each 10‐point increase in CVH was associated with 27.9% (HR: 0.721; 95% CI: 0.654, 0.795), 25.6% (HR: 0.744; 95% CI: 0.712, 0.778), 21.9% (HR: 0.781; 95% CI: 0.757, 0.806), 17.7% (HR: 0.823; 95% CI: 0.798, 0.849), and 11.9% (HR: 0.881; 95% CI: 0.842, 0.922) lower risks of T2D onset at ages < 40, 40–50, 50–60, 60–70, and ≥ 70 years, respectively. When participants were divided into low, intermediate, and high CVH groups, the results were similar. A significant multiplicative interaction was observed between CVH and age groups (*P*_interaction_ < 0.001). Among the various CVH components, improvements in scores of nicotine exposure, BMI, blood pressure, and lipid contributed more significantly to risk reduction of young‐onset T2D (Table S5). Stratification by family history revealed that the multiplicative interaction between CVH and age groups persisted in both populations with and without family history (*P*_interaction_ < 0.001). More importantly, the risk‐lowering impact of higher CVH on young‐onset T2D was stronger among individuals with family history. For instance, for a 10‐point increase in CVH, the HR (95% CI) of developing T2D at the ages of < 40, 40–50, and 50–60 years was 0.642 (0.555, 0.743), 0.683 (0.640, 0.729), and 0.747 (0.712, 0.784) in the population with family history, respectively, compared to 0.797 (0.695, 0.913), 0.807 (0.759, 0.858), and 0.811 (0.778, 0.846) in those without family history (Table 2). The interaction between CVH and age groups was observed in both men and women (*P*_interaction_ < 0.001) (Table S6).

**TABLE 2:** The association of CVH score with the incidence risk of T2D by onset age of T2D and family history.

Onset age groups	CVH level, HR (95% CI)			
	Per 10 points increase	Low (< 50)	Intermediate (50–80)	High (≥ 80)
Total				
< 40 years	0.721 (0.654, 0.795)	1.000 (Ref.)	0.435 (0.280, 0.677)	0.299 (0.182, 0.491)
40–50 years	0.744 (0.712, 0.778)	1.000 (Ref.)	0.458 (0.383, 0.547)	0.316 (0.256, 0.392)
50–60 years	0.781 (0.757, 0.806)	1.000 (Ref.)	0.605 (0.532, 0.687)	0.358 (0.304, 0.420)
60–70 years	0.823 (0.798, 0.849)	1.000 (Ref.)	0.701 (0.624, 0.788)	0.401 (0.341, 0.470)
≥ 70 years	0.881 (0.842, 0.922)	1.000 (Ref.)	0.832 (0.712, 0.972)	0.571 (0.454, 0.719)
*P*_interation_	< 0.001			
Family history				
< 40 years	0.642 (0.555, 0.743)	1.000 (Ref.)	0.375 (0.213, 0.661)	0.184 (0.091, 0.375)
40–50 years	0.683 (0.640, 0.729)	1.000 (Ref.)	0.432 (0.340, 0.549)	0.220 (0.160, 0.301)
50–60 years	0.747 (0.712, 0.784)	1.000 (Ref.)	0.536 (0.447, 0.643)	0.276 (0.216, 0.354)
60–70 years	0.826 (0.784, 0.871)	1.000 (Ref.)	0.740 (0.610, 0.897)	0.423 (0.321, 0.557)
≥ 70 years	0.933 (0.855, 1.019)	1.000 (Ref.)	0.744 (0.562, 0.986)	0.848 (0.564, 1.276)
*P*_interation_	< 0.001			
No family history				
< 40 years	0.797 (0.695, 0.913)	1.000 (Ref.)	0.534 (0.253, 1.128)	0.442 (0.200, 0.977)
40–50 years	0.807 (0.759, 0.858)	1.000 (Ref.)	0.513 (0.391, 0.673)	0.430 (0.316, 0.584)
50–60 years	0.811 (0.778, 0.846)	1.000 (Ref.)	0.699 (0.582, 0.838)	0.447 (0.360, 0.557)
60–70 years	0.823 (0.792, 0.855)	1.000 (Ref.)	0.680 (0.587, 0.787)	0.394 (0.324, 0.479)
≥ 70 years	0.865 (0.821, 0.912)	1.000 (Ref.)	0.877 (0.727, 1.057)	0.510 (0.386, 0.674)
*P*_interation_	< 0.001			

*Note:* Models were adjusted for sex, living region, urban area, education level, alcohol drinking status, family history of T2D (not included in the model stratified by family history), and subcohorts. *P*_interaction_ represented the significance of interaction effect between CVH and age groups.

Abbreviations: 95% CI, 95% confidence interval; CVH, cardiovascular health; HR, hazard ratio; T2D, type 2 diabetes.

**FIGURE 3:**
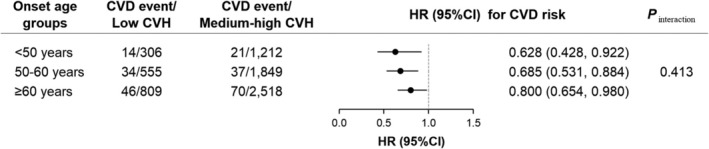
The association of CVH score with CVD risk among T2D patients with different onset age. 95% CI, 95% confidence interval; CVD, cardiovascular disease; CVH, cardiovascular health; HR, hazard ratio; T2D, type 2 diabetes. HRs (95% CIs) for CVD risk associated with high CVH across three age groups were calculated using Cox regression models, with the population with low CVH serving as the reference group. Models were adjusted for age, sex, living region, urban area, education level, alcohol drinking status, family history of CVD, and subcohorts. *P*_interaction_ represented the significance of interaction effect between CVH and age groups.

Moreover, among individuals with T2D, medium–high CVH was associated with 37.2% (HR: 0.628; 95% CI: 0.428, 0.922), 31.5% (HR: 0.685; 95% CI: 0.531, 0.884), and 20.0% (HR: 0.800; 95% CI: 0.654, 0.980) lower risks of CVD in those who developed T2D at < 50, 50–60, and ≥ 60 years, respectively, compared to low CVH counterparts. No significant interaction was observed between CVH and age groups (*P*_interaction_ = 0.413) (Figure 3). The results of the associations between each CVH component and CVD risk are shown in Figure S5. Subgroup analyses indicated similar results for both males and females (Figure S6).

Similar results were observed in the sensitivity analyses through the competing risk model, showing the stability of the results (Figures S7 and S8 and Tables S7 and S8).

## 4 Discussion

Overall, our findings demonstrated that the association between family history of T2D and T2D risk was more pronounced among individuals with younger onset ages, emphasizing that individuals with better CVH can mitigate this risk, especially in those with family history. Furthermore, building upon the association between young onset age of T2D and increased CVD risk, we found the association stronger in individuals with family history of T2D, while good CVH also alleviates the CVD risk posed by young‐onset T2D. Our findings highlighted that family history of T2D and young‐onset T2D were important characteristics for identifying high‐risk individuals for CVD, with good CVH potentially delaying T2D onset and reducing related CVD risk.

Current diabetes prevention guidelines and recommendations mainly target reducing diabetes incidence, with limited emphasis on identifying risk factors and interventions to delay onset [6, 26]. Although family history is a known risk factor, large‐scale prospective evidence quantifying its impact on onset age has been scarce [9, 27, 28, 29]. Our longitudinal study demonstrated that family history significantly increased the risk of developing young‐onset T2D. Notably, the significant interaction between family history and age group indicated that the detrimental impact of familial predisposition was not uniform across the lifespan but was strongest at younger ages. This supported a risk‐stratified clinical approach, where individuals with a family history should be prioritized for intensive prevention efforts early in adulthood, before the age at which routine T2D screening is typically initiated. This association may be attributed mainly to shared genetic variants associated with early‐onset diabetes among families, including *TCF7L2*, *MC4R*, *CDC123*, *KCNQ1*, *IGFBP2*, *SLC16A11*, and *PHF2* [30]. Furthermore, poor socioeconomic status and lifestyle factors often found within families may also contribute to early‐onset T2D [6].

An increasing number of studies recognized the detrimental impact of young‐onset T2D, revealing links between T2D onset age and adverse outcomes like CVD, dementia, and cancer across different countries [12, 31, 32, 33]. However, no previous study has investigated how family history impacts the relationship between T2D onset age and incident CVD. Our findings show that among participants with a family history of T2D, young‐onset T2D significantly increases CVD risk compared to those without such a history. This is a novel insight that expands the understanding of familial susceptibility on subsequent CVD events among diabetic patients. The elevated CVD risk linked to young‐onset diabetes can be attributed to longer exposure to suboptimal blood glucose levels in younger patients and complications such as poor blood glucose control, β‐cell dysfunction, and insulin resistance [34, 35, 36, 37].

Family history is an unmodifiable risk factor, and efforts should focus on promoting behavioral changes to mitigate inherent risks [38]. Studies evaluating the interplay of family history and lifestyle on T2D onset age remain limited and yield conflicting results [8, 39, 40]. For instance, the Joint Asia Diabetes Evaluation (JADE) registry study found that adopting a healthy lifestyle can delay diabetes onset associated with family history [8], while another cross‐sectional study in the United States observed that the risk of early‐onset T2D with family history was greater in leaner individuals [39]. Additionally, a study from Japan indicated that the association between family history and diabetes is not affected by lifestyle [40]. These inconsistent findings may arise from the limitations of cross‐sectional studies and genetic variations among different ethnic groups. Hence, it is essential to evaluate the relationships among family history, lifestyle, and T2D onset age more comprehensively within the Chinese population using prospective data. Our study incorporates additional factors such as sleep, BP, and lipid levels, following AHA recommendations [16], to provide a thorough assessment of CVH in mitigating the risks of young‐onset T2D related to family history. The significant interaction between CVH and age group, with a stronger protective effect against T2D at younger ages, carried important clinical implications. It suggested that improving and maintaining ideal CVH is particularly crucial for younger individuals, especially those with family history, as it may attenuate their familial susceptibility to early disease onset. This finding reinforced the value of early‐life promotion of CVH as a potent preventive strategy.

Although reducing T2D incidence and delaying its onset is desirable, early intervention remains crucial for individuals who already have T2D, especially those who develop it early. Current evidence supporting management strategies for young diabetic patients mainly stems from trials involving older patients, highlighting a lack of focus on younger populations [6]. Previous trials for young patients have primarily addressed dietary intervention and weight reduction, demonstrating benefits for lifestyle interventions on glucose control and weight management [41, 42, 43]. However, the effects of these interventions on subsequent cardiovascular events remain unclear. Our study, based on real‐world observations, explores the interactions between family history and various lifestyle and metabolic factors, clarifying how these collectively influence the risk of young‐onset T2D and CVD. Future research could delve into the effectiveness of early screening and intervention strategies to identify how best to reduce the risks of T2D and CVD in vulnerable populations.

Our results carry implications for public health strategy and clinical practice. First, they support establishing or enhancing national screening programs that target younger adults (e.g., < 40 years) with family history of T2D. Given the markedly elevated risk in this group, incorporating family history into screening algorithms could lead to earlier detection and intervention. Second, primary care should be updated to integrate assessment of both T2D family history and CVH metrics during clinical encounters for adults, particularly the young. This would facilitate risk stratification and personalized counseling. Third, for individuals identified as high‐risk, our findings advocate for structured early intervention models that aggressively promote ideal CVH. Specifically, the management of factors such as BP, blood glucose, and lipid metabolism may be crucial. Such models should focus on achieving and maintaining optimal cardiovascular metrics from a young age to delay or prevent T2D onset. For those already diagnosed with young‐onset T2D, intensive CVH management is crucial to mitigate their disproportionately high CVD risk.

To our knowledge, this research is the first large‐scale prospective cohort study quantitatively assessing how family history and CVH affect the onset age of T2D and the subsequent link between T2D onset age and CVD incidence. The study's design facilitated the reliable collection of data on new‐onset T2D and CVD outcomes. However, we acknowledge certain limitations. Firstly, our study did not include T2D diagnosis through postprandial blood glucose, the 75 g oral glucose tolerance test, or glycated hemoglobin. Nonetheless, the FBG test we utilized is a straightforward and reliable diagnostic criterion for T2D commonly employed in clinical settings, and the underestimation of T2D suggested that our study results tend to be conservative. Secondly, while individual lifestyles can fluctuate, our sample size was insufficient to analyze the effects of lifestyle modifications, which warrants further study. Thirdly, information on family history and lifestyle was self‐reported, potentially introducing recall and misclassification bias. Additionally, self‐reported lifestyle data may be subject to social desirability bias. This bias may dilute the observed associations toward the null, suggesting that our risk estimates may be conservative. Fourthly, this study did not include direct genetic data, and the observed associations between family history and the outcomes could be partly confounded by unmeasured genetic factors, which future studies incorporating genetic information could help disentangle. Lastly, despite appropriate adjustments, the influence of unmeasured confounding factors on outcomes cannot be entirely ruled out.

## 5 Conclusions

In conclusion, family history serves as a crucial risk factor for the early onset of T2D, amplifying the detrimental impact of young‐onset T2D on the heightened risk of CVD. Effective CVH management holds the potential to postpone the onset of T2D and reduce the risk of subsequent CVD. These findings support the consideration of the significance of onset age and family predisposition in the management of T2D and CVD, advocating the need for risk reduction through lifestyle modifications and holistic management of metabolic levels in patients.

## Funding

This work was supported by the National Science and Technology Major Program for Noncommunicable Chronic Diseases (2023ZD0503500), the National Natural Science Foundation of China (82322059), the Chinese Academy of Medical Sciences (CAMS) (10.13039/501100005150) Innovation Fund for Medical Sciences (2021‐I2M‐1‐010), the National Natural Science Foundation of China (82030102, 82330106, 82473720), and the National High Level Hospital Clinical Research Funding (2022‐GSP‐GG‐1, 2022‐GSP‐GG‐2).

## Disclosure

The authors have nothing to report.

## Conflicts of Interest

The authors declare no conflicts of interest.

## Supporting information


**Table S1:** Measurement and definition of CVH metric.
**Table S2:** Baseline characteristics of participants stratified by family history.
**Table S3:** Study characteristics of patients with T2D and their matched controls.
**Table S4:** The association of family history with the incidence risk of T2D by onset age of T2D in different sex groups.
**Table S5:** The associations of CVH components with the incidence risk of T2D by onset age of T2D.
**Table S6:** The association of CVH score with the incidence risk of T2D by onset age of T2D in different sex groups.
**Table S7:** The association of family history with the incidence risk of T2D by onset age of T2D using competing risk model.
**Table S8:** The association of CVH score with the incidence risk of T2D by onset age of T2D and family history using competing risk model.
**Figure S1:** The research concept and process.
**Figure S2:** Flow diagram of the study population for the analyses.
**Figure S3:** The differences in the onset age of T2D among population with different family history status and CVH level.
**Figure S4:** The association of T2D with different onset age with CVD risk among males (A) and females.
**Figure S5:** The association of CVH components with CVD risk among T2D patients with different onset age.
**Figure S6:** The association of CVH score with CVD risk among (A) male and (B) female patients with T2D with different onset age.
**Figure S7:** The association of T2D with different onset age with CVD risk among total population (A), individuals with (B) and without (C) family history using competing risk model.
**Figure S8:** The association of CVH score with CVD risk among T2D patients with different onset age using competing risk model.

## Data Availability

Research data are not shared.
