# Supplementary material for: Family History and Adherence to Ideal Cardiovascular Health: Counteracting Impacts on the Onset Age of Type 2 Diabetes and Subsequent Cardiovascular Disease Risk
Source: J Diabetes. 2026 Aug 5;18(8):e70256. doi: 10.1111/1753-0407.70256 (PMC13441548; doi:10.1111/1753-0407.70256)
Supplement: Supplementary file 1 [file JDB-18-e70256-s001.docx]

Supplemental information

**Family History and Adherence to Ideal Cardiovascular Health: Counteracting Impacts on the Onset Age of Type 2 Diabetes and Subsequent Cardiovascular Disease Risk**

**Table S1** Measurement and definition of CVH metric

**Table S2** Baseline characteristics of participants stratified by family history

**Table S3** Study characteristics of patients with T2D and their matched controls

**Table S4** The association of family history with the incidence risk of T2D by onset age of T2D in different sex groups

**Table S5** The associations of CVH components with the incidence risk of T2D by onset age of T2D

**Table S6** The association of CVH score with the incidence risk of T2D by onset age of T2D in different sex groups

**Table S7** The association of family history with the incidence risk of T2D by onset age of T2D using competing risk model

**Table S8** The association of CVH score with the incidence risk of T2D by onset age of T2D and family history using competing risk model

**Figure S1** The research concept and process

**Figure S2** Flow diagram of the study population for the analyses

**Figure S3** The differences in the onset age of T2D among population with different family history status and CVH level

**Figure S4** The association of T2D with different onset age with CVD risk among males (A) and females

**Figure S5** The association of CVH components with CVD risk among T2D patients with different onset age

**Figure S6** The association of CVH score with CVD risk among (A) male and (B) female patients with T2D with different onset age

**Figure S7** The association of T2D with different onset age with CVD risk among total population (A), individuals with (B) and without (C) family history using competing risk model

**Figure S8** The association of CVH score with CVD risk among T2D patients with different onset age using competing risk model

Table S1 Measurement and definition of CVH metric

| CVH individual metric | Method of measurement | Quantification of metric | | |
| --- | --- | --- | --- | --- |
| Diet health | Self-reported intake and frequencies of fruits and vegetables, fish, soybean products, red meat, and tea were collected via a standard food frequency questionnaire. | Points | | Diet component |
|  |  | 20 | | Fruits and vegetables ≥500 g/day |
|  |  | 20 | | Fish ≥200g/week |
|  |  | 20 | | Soybean products ≥125 g/day |
|  |  | 20 | | Red meat <75 g/day |
|  |  | 20 | | Tea ≥50 g/month |
|  |  | The final score of diet health is the sum of the five component scores. | | |
| Physical activity | Self-reported minutes of moderate or vigorous physical activity per week were collected via a standard questionnaire.  1 minute of vigorous physical activity is equivalent to 2 minutes of moderate physical activity. | Points | | Moderate or vigorous activity per week |
|  |  | 100 | | ≥150 minutes |
|  |  | 90 | | 120-149 minutes |
|  |  | 80 | | 90-119 minutes |
|  |  | 60 | | 60-89 minutes |
|  |  | 40 | | 30-59 minutes |
|  |  | 20 | | 1-29 minutes |
|  |  | 0 | | 0 |
| Nicotine exposure | Self-reported smoking status and age of smoking cessation were collected via a standard questionnaire by asking “Have you smoked at least 20 packs of cigarettes in your lifetime?” and “Are you smoking currently?”. | Points | | Smoking status |
|  |  | 100 | | Never smoker |
|  |  | 75 | | Former smokers quit ≥5 year |
|  |  | 50 | | Former smokers quit 1 - <5 year |
|  |  | 25 | | Former smokers quit <1 year |
|  |  | 0 | | Current smoker |
| Sleep duration | Self-reported average hours of sleep per night were collected via a standard questionnaire by asking “On average, how many hours of sleep do you get per night in the last year? | Points | | Average hours of sleep per night |
|  |  | 100 | | 7 - <9 hours |
|  |  | 90 | | 9 - <10 hours |
|  |  | 70 | | 6 - <7 hours |
|  |  | 40 | | 5 - <6 or ≥10 hours |
|  |  | 20 | | 4 - <5 hours |
|  |  | 0 | | <4 hours |
| Body mass index | Body mass index was calculated as weight in kilograms divided by height in meters squared. | Points | | Body mass index |
|  |  | 100 | | <23 kg/m^2^ |
|  |  | 75 | | 23.0-24.9 kg/m^2^ |
|  |  | 50 | | 25.0-29.9 kg/m^2^ |
|  |  | 25 | | 30.0-34.9 kg/m^2^ |
|  |  | 0 | | ≥35.0 kg/m^2^ |
| Blood pressure | Blood pressure was measured by standardized mercury sphygmomanometer or electronic sphygmomanometer. | Points | | Systolic and diastolic blood pressure |
|  |  | 100 | | <120/<80 |
|  |  | 75 | | 120-129/<80 |
|  |  | 50 | | 130-139 or 80-89 |
|  |  | 25 | | 140-159 or 90-99 |
|  |  | 0 | | ≥160 or ≥100 |
|  |  | If drug-treated level, subtract 20 points. | | |
| Blood lipid | Serum cholesterol was measured enzymatically.  Non-HDLC was calculated by total cholesterol minus HDLC. | Points | | Non-HDLC |
|  |  | 100 | | <130 mg/dL |
|  |  | 60 | | 130-159 mg/dL |
|  |  | 40 | | 160-189 mg/dL |
|  |  | 20 | | 190-219 mg/dL |
|  |  | 0 | | ≥220 mg/dL |
|  |  | If drug-treated level, subtract 20 points. | | |
| Blood glucose | Fasting blood glucose was measured by glucose oxidase method. | Points | Fasting blood glucose | |
|  |  | 100 | No history of diabetes with FBG <100 mg/dL | |
|  |  | 60 | No diabetes with FBG 100-125 mg/dL | |
|  |  | 40 | Diabetes with FBG 126-154 mg/dL | |
|  |  | 30 | Diabetes with FBG 155-183 mg/dL | |
|  |  | 20 | Diabetes with FBG 184-210 mg/dL | |
|  |  | 10 | Diabetes with FBG 211-239 mg/dL | |
|  |  | 0 | Diabetes with FBG ≥240 mg/dL | |

Abbreviations: CVH, cardiovascular health; HDLC, high-density lipoprotein cholesterol.

Table S2 Baseline characteristics of participants stratified by family history

| Characteristics | Total | Family history | No family history | *P* value |
| --- | --- | --- | --- | --- |
| Participants, N (%) | 79,831 (100.0) | 12,028 (15.1) | 67,803 (84.9) |  |
| Age, years | 51.5±11.7 | 50.5±10.9 | 51.6±11.8 | <0.001 |
| Male, N (%) | 31,724 (39.7) | 4,653 (38.7) | 27,071 (39.9) | 0.011 |
| Northern region, N (%) | 41,937 (52.5) | 7,465 (62.1) | 34,472 (50.8) | <0.001 |
| Urban area, N (%) | 7,658 (9.6) | 2,005 (16.7) | 5,653 (8.3) | <0.001 |
| High school education or above, N (%) | 10,616 (13.4) | 2,180 (18.2) | 8,436 (12.5) | <0.001 |
| Family history of CVD, N (%) | 8,536 (10.7) | 1,837 (15.3) | 6,699 (9.9) | <0.001 |
| CVH score | 69.2±13.4 | 67.4±13.8 | 69.5±13.3 | <0.001 |
| Current smokers, N (%) | 17,390 (21.9) | 2,593 (21.6) | 14,797 (21.9) | 0.605 |
| Alcohol drinkers, N (%) | 14,404 (18.1) | 2,147 (17.9) | 12,257 (18.1) | 0.586 |
| Ideal physical activity, N (%) | 54,817 (68.8) | 7,907 (65.9) | 46,910 (69.3) | <0.001 |
| Ideal diet habits, N (%) | 41,407 (57.4) | 6,389 (61.6) | 35,018 (56.7) | <0.001 |
| Ideal sleep duration, N (%) | 54,586 (68.4) | 7,994 (66.5) | 46,592 (68.7) | <0.001 |
| Overweight, N (%) | 36,103 (45.3) | 6,689 (55.6) | 29,414 (43.4) | <0.001 |
| Systolic blood pressure, mmHg | 129.0±21.4 | 130.3±22.2 | 128.8±21.3 | <0.001 |
| Diastolic blood pressure, mmHg | 79.5±11.7 | 80.9±11.7 | 79.2±11.7 | <0.001 |
| Fasting blood glucose, mg/dL | 91.1±23.4 | 97.8±33.1 | 89.9±21.0 | <0.001 |
| Total cholesterol, mg/dL | 175.9±35.0 | 179.7±35.5 | 175.6±34.8 | <0.001 |
| LDLC, mg/dL | 97.1±30.2 | 99.8±31.0 | 96.6±30.0 | <0.001 |
| HDLC, mg/dL | 51.2±12.8 | 49.6±12.5 | 51.5±12.8 | <0.001 |

Abbreviations: CVD, cardiovascular disease; CVH, cardiovascular health; LDLC, low-density lipoprotein cholesterol; HDLC, high-density lipoprotein cholesterol.

Characteristics were presented as the mean±standard deviation for continuous variables and frequency (percentage) for categorical variables.

Table S3 Study characteristics of patients with T2D and their matched controls

| Characteristics | Overall | T2D patients | Matched controls | *P* value^a^ | Onset age of new-onset T2D patients | | | |
| --- | --- | --- | --- | --- | --- | --- | --- | --- |
|  |  |  |  |  | <50 years | 50-60 years | ≥60 years | *P* value^b^ |
| Participants, N | 14,774 | 7,387 | 7,387 |  | 1,547 | 2,452 | 3,388 |  |
| Age, years | 52.9±10.3 | 52.9±10.3 | 52.9±10.3 | 0.970 | 39.6±5.1 | 49.5±4.5 | 61.5±6.3 | <0.001 |
| Male, N (%) | 5,826 (39.4) | 2,913 (39.4) | 2,913 (39.4) | 1.000 | 644 (41.6) | 946 (38.6) | 1,323 (39.1) | 0.130 |
| Northern region, N (%) | 8,062 (54.6) | 4,031 (54.6) | 4,031 (54.6) | 1.000 | 972 (62.8) | 1,343 (54.8) | 1,716 (50.7) | <0.001 |
| Urban area, N (%) | 1,792 (12.1) | 871 (11.7) | 921 (12.5) | 0.217 | 201 (13.0) | 356 (14.5) | 314 (9.3) | <0.001 |
| High school education or above, N (%) | 2,023(13.7) | 955 (12.9) | 1,068 (14.5) | 0.007 | 323 (20.9) | 411 (16.8) | 221 (6.5) | <0.001 |
| Family history of CVD, N (%) | 1,900 (12.9) | 844 (11.4) | 1,056 (14.3) | 0.007 | 204 (13.2) | 338 (13.8) | 302 (8.9) | <0.001 |
| CVH score | 63.2±12.6 | 58.5±11.7 | 67.8±11.7 | <0.001 | 59.9±12.4 | 58.4±11.6 | 57.9±11.4 | <0.001 |
| Current smokers, N (%) | 2,796 (18.9) | 1,292 (17.5) | 1,504 (20.4) | <0.001 | 363 (23.5) | 459 (18.7) | 470 (13.9) | <0.001 |
| Alcohol drinkers, N (%) | 2,066 (14.9) | 1,047 (15.1) | 1,019 (14.6) | 0.413 | 268 (18.3) | 385 (16.8) | 394 (12.5) | <0.001 |
| Hypertension, N (%) | 7,423 (50.2) | 4,217 (57.1) | 3,206 (43.4) | <0.001 | 681 (44.0) | 1,350 (55.1) | 2,186 (64.5) | <0.001 |
| Dyslipidemia, N (%) | 6,226 (42.1) | 3,557 (48.2) | 2,669 (36.1) | <0.001 | 767 (49.6) | 1,263 (51.5) | 1,527 (45.1) | <0.001 |
| BMI, kg/m^2^ | 25.0±3.7 | 25.5±3.7 | 24.5±3.5 | <0.001 | 25.8±3.8 | 25.8±3.7 | 25.2±3.7 | <0.001 |
| Systolic blood pressure, mmHg | 135.0±19.3 | 138.1±19.3 | 131.9±18.7 | <0.001 | 131.8±16.8 | 136.8±18.7 | 142.1±20.0 | <0.001 |
| Diastolic blood pressure, mmHg | 80.8±10.7 | 82.1±10.6 | 79.4±10.7 | <0.001 | 82.9±10.4 | 82.7±10.5 | 81.4±10.9 | <0.001 |
| Fasting blood glucose, mg/dL | 120.7±45.1 | 149.6±46.9 | 91.7±13.7 | <0.001 | 154.4±51.5 | 150.8±49.2 | 146.6±42.6 | <0.001 |
| Total cholesterol, mg/dL | 189.3±39.4 | 193.8±41.2 | 184.7±36.9 | <0.001 | 191.9±41.3 | 194.1±41.7 | 194.4±40.8 | 0.109 |
| LDLC, mg/dL | 106.0±33.9 | 107.2±35.7 | 104.9±32.2 | <0.001 | 104.5±34.0 | 107.0±36.6 | 108.5±35.7 | 0.003 |
| HDLC, mg/dL | 53.0±18.0 | 52.9±19.0 | 53.2±16.9 | 0.277 | 51.6±19.1 | 52.0±18.6 | 54.1±19.3 | <0.001 |

Abbreviations: T2D, type 2 diabetes; CVH, cardiovascular health; LDLC, low-density lipoprotein cholesterol; HDLC, high-density lipoprotein cholesterol

^a^ *P* value represented differences between the groups of T2D patients and matched controls.

^b^ *P* value represented differences among T2D patients with 3 onset age groups.

Table S4 The association of family history with the incidence risk of T2D by onset age of T2D in different sex groups

| Onset age groups | Male, HR (95%CI) | Female, HR (95%CI) |
| --- | --- | --- |
| **<40 years** |  |  |
| No family history | 1.000 (Ref.) | 1.000 (Ref.) |
| Family history | 3.851 (2.667, 5.561) | 4.175 (2.951, 5.906) |
| **40-50 years** |  |  |
| No family history | 1.000 (Ref.) | 1.000 (Ref.) |
| Family history | 3.844 (3.222, 4.587) | 3.697 (3.198, 4.274) |
| **50-60 years** |  |  |
| No family history | 1.000 (Ref.) | 1.000 (Ref.) |
| Family history | 3.779 (3.487, 4.097) | 3.542 (3.188, 3.936) |
| **60-70 years** |  |  |
| No family history | 1.000 (Ref.) | 1.000 (Ref.) |
| Family history | 3.822 (3.526, 4.142) | 3.553 (3.195, 3.950) |
| **≥70 years** |  |  |
| No family history | 1.000 (Ref.) | 1.000 (Ref.) |
| Family history | 3.270 (2.981, 3.587) | 2.898 (2.458, 3.416) |
| ***P*_interaction_** | 0.076 | 0.002 |

Abbreviations: T2D, type 2 diabetes; HR, hazard ratio; 95%CI, 95% confidence interval; BMI, body mass index.

Model was adjusted for living region, urban area, education level, current smoking status, alcohol drinking status, physical activity, BMI, hypertension, dyslipidemia, and subcohorts.

*P*_interaction_ represented the significance of interaction effect between family history and age groups.

Table S5 The associations of CVH components with the incidence risk of T2D by onset age of T2D

|  | Per 10 points increase, HR (95%CI) | *P*_interation_ |
| --- | --- | --- |
| **Diet health** |  |  |
| <40 years | 0.910 (0.849, 0.976) | 0.553 |
| 40-50 years | 1.008 (0.978, 1.040) |  |
| 50-60 years | 0.985 (0.964, 1.007) |  |
| 60-70 years | 0.978 (0.956, 1.000) |  |
| ≥70 years | 1.005 (0.971, 1.040) |  |
| **Physical activity** |  |  |
| <40 years | 0.977 (0.951, 1.005) | 0.148 |
| 40-50 years | 0.986 (0.973, 0.999) |  |
| 50-60 years | 0.995 (0.985, 1.004) |  |
| 60-70 years | 1.001 (0.992, 1.010) |  |
| ≥70 years | 1.003 (0.991, 1.015) |  |
| **Nicotine exposure** |  |  |
| <40 years | 0.905 (0.872, 0.938) | 0.003 |
| 40-50 years | 0.951 (0.934, 0.969) |  |
| 50-60 years | 0.961 (0.948, 0.973) |  |
| 60-70 years | 0.985 (0.973, 0.997) |  |
| ≥70 years | 1.003 (0.986, 1.021) |  |
| **Sleep duration** |  |  |
| <40 years | 0.981 (0.923, 1.042) | 0.219 |
| 40-50 years | 0.984 (0.958, 1.011) |  |
| 50-60 years | 0.994 (0.975, 1.014) |  |
| 60-70 years | 0.999 (0.981, 1.017) |  |
| ≥70 years | 1.014 (0.989, 1.039) |  |
| **Body mass index** |  |  |
| <40 years | 0.863 (0.823, 0.904) | <0.001 |
| 40-50 years | 0.844 (0.826, 0.863) |  |
| 50-60 years | 0.837 (0.823, 0.851) |  |
| 60-70 years | 0.865 (0.851, 0.879) |  |
| ≥70 years | 0.897 (0.877, 0.918) |  |
| **Blood pressure** |  |  |
| <40 years | 0.885 (0.853, 0.920) | <0.001 |
| 40-50 years | 0.921 (0.906, 0.936) |  |
| 50-60 years | 0.935 (0.924, 0.946) |  |
| 60-70 years | 0.942 (0.931, 0.953) |  |
| ≥70 years | 0.956 (0.940, 0.973) |  |
| **Blood lipid** |  |  |
| <40 years | 0.894 (0.850, 0.940) | <0.001 |
| 40-50 years | 0.897 (0.879, 0.915) |  |
| 50-60 years | 0.917 (0.905, 0.931) |  |
| 60-70 years | 0.921 (0.908, 0.933) |  |
| ≥70 years | 0.945 (0.926, 0.966) |  |

Abbreviations: CVH, cardiovascular health; T2D, type 2 diabetes; HR, hazard ratio; 95%CI, 95% confidence interval.

Models were adjusted for sex, living region, urban area, education level, alcohol drinking status, family history of T2D (not included in the model stratified by family history), and subcohorts.

*P*_interaction_ represented the significance of interaction effect between CVH components and age groups.

Table S6 The association of CVH score with the incidence risk of T2D by onset age of T2D in different sex groups

| Onset age groups | CVH level, HR (95%CI) | | | |
| --- | --- | --- | --- | --- |
|  | Per 10 points increase | Low (<50) | Intermediate (50-80) | High (≥80) |
| **Male** |  |  |  |  |
| <40 years | 0.782 (0.686, 0.893) | 1.000 (Ref.) | 0.477 (0.219, 0.782) | 0.467 (0.254, 0.858) |
| 40-50 years | 0.739 (0.693, 0.789) | 1.000 (Ref.) | 0.483 (0.393, 0.595) | 0.377 (0.270, 0.528) |
| 50-60 years | 0.802 (0.764, 0.842) | 1.000 (Ref.) | 0.593 (0.504, 0.697) | 0.466 (0.361, 0.602) |
| 60-70 years | 0.825 (0.787, 0.865) | 1.000 (Ref.) | 0.602 (0.514, 0.706) | 0.398 (0.309, 0.511) |
| ≥70 years | 0.900 (0.840, 0.964) | 1.000 (Ref.) | 0.828 (0.654, 1.047) | 0.593 (0.412, 0.852) |
| *P*_interation_ | <0.001 | | | |
| **Female** | | | | |
| <40 years | 0.661 (0.569, 0.767) | 1.000 (Ref.) | 0.256 (0.093, 0.707) | 0.148 (0.053, 0.415) |
| 40-50 years | 0.751 (0.705, 0.799) | 1.000 (Ref.) | 0.411 (0.284, 0.595) | 0.275 (0.187, 0.403) |
| 50-60 years | 0.767 (0.735, 0.801) | 1.000 (Ref.) | 0.643 (0.516, 0.800) | 0.348 (0.273, 0.444) |
| 60-70 years | 0.819 (0.785, 0.853) | 1.000 (Ref.) | 0.834 (0.698, 0.997) | 0.442 (0.354, 0.551) |
| ≥70 years | 0.871 (0.820, 0.925) | 1.000 (Ref.) | 0.846 (0.686, 1.043) | 0.569 (0.421, 0.770) |
| *P*_interation_ | <0.001 | | | |

Abbreviations: CVH, cardiovascular health; T2D, type 2 diabetes; HR, hazard ratio; 95%CI, 95% confidence interval.

Models were adjusted for living region, urban area, education level, alcohol drinking status, family history of T2D (not included in the model stratified by family history), and subcohorts.

*P*_interaction_ represented the significance of interaction effect between CVH and age groups.

Table S7 The association of family history with the incidence risk of T2D by onset age of T2D using competing risk model

| Onset age groups | HR (95%CI) | | *P*_interaction_ |
| --- | --- | --- | --- |
|  | No family history | Family history |  |
| <40 years | 1.000 (Ref.) | 3.925 (3.049, 5.050) | 0.026 |
| 40-50 years | 1.000 (Ref.) | 3.898 (3.494, 4.349) |  |
| 50-60 years | 1.000 (Ref.) | 3.741 (3.445, 4.062) |  |
| 60-70 years | 1.000 (Ref.) | 3.685 (3.387, 4.010) |  |
| ≥70 years | 1.000 (Ref.) | 3.355 (2.937, 3.833) |  |

Abbreviations: T2D, type 2 diabetes; HR, hazard ratio; 95%CI, 95% confidence interval.

Model was adjusted for sex, living region, urban area, education level, current smoking status, alcohol drinking status, physical activity, BMI, hypertension, dyslipidemia, and subcohorts .

*P*_interaction_ represented the significance of interaction effect between family history and age groups.

Table S8 The association of CVH score with the incidence risk of T2D by onset age of T2D and family history using competing risk model

| Onset age groups | CVH level, HR (95%CI) | | | |
| --- | --- | --- | --- | --- |
|  | Per 10 points | Low (<50) | Intermediate (50-80) | High (≥80) |
| **Total** |  |  |  |  |
| <40 years | 0.680 (0.614, 0.753) | 1.000 (Ref.) | 0.368 (0.234, 0.578) | 0.227 (0.136, 0.378) |
| 40-50 years | 0.712 (0.380, 0.746) | 1.000 (Ref.) | 0.429 (0.358, 0.514) | 0.270 (0.217, 0.335) |
| 50-60 years | 0.767 (0.744, 0.792) | 1.000 (Ref.) | 0.585 (0.514, 0.666) | 0.330 (0.281, 0.389) |
| 60-70 years | 0.815 (0.790, 0.841) | 1.000 (Ref.) | 0.692 (0.615, 0.779) | 0.387 (0.329, 0.455) |
| ≥70 years | 0.867 (0.829, 0.906) | 1.000 (Ref.) | 0.824 (0.705, 0.963) | 0.530 (0.420, 0.669) |
| *P*_interation_ | <0.001 | | | |
| **Family history** |  |  |  |  |
| <40 years | 0628 (0.544, 0.724) | 1.000 (Ref.) | 0.360 (0.204, 0.635) | 0.160 (0.078, 0.330) |
| 40-50 years | 0.666 (0.625, 0.711) | 1.000 (Ref.) | 0.414 (0.326, 0.527) | 0.201 (0.147, 0.276) |
| 50-60 years | 0.742 (0.708, 0.778) | 1.000 (Ref.) | 0.531 (0.441, 0.639) | 0.269 (0.209, 0.345) |
| 60-70 years | 0.825 (0.783, 0.868) | 1.000 (Ref.) | 0.744 (0.615, 0.900) | 0.421 (0.320, 0.553) |
| ≥70 years | 0.936 (0.857, 1.021) | 1.000 (Ref.) | 0.764 (0.578, 1.010) | 0.859 (0.570, 1.290) |
| *P*_interation_ | <0.001 | | | |
| **No family history** | | | | |
| <40 years | 0.726 (0.628, 0.840) | 1.000 (Ref.) | 0.401 (0.189, 0.850) | 0.293 (0.131, 0.653) |
| 40-50 years | 0.762 (0.715, 0.813) | 1.000 (Ref.) | 0.484 (0.368, 0.636) | 0.350 (0.256, 0.479) |
| 50-60 years | 0.787 (0.755, 0.821) | 1.000 (Ref.) | 0.658 (0.549, 0.789) | 0.396 (0.317, 0.493) |
| 60-70 years | 0.811 (0.780, 0.842) | 1.000 (Ref.) | 0.661 (0.571, 0.765) | 0.372 (0.305, 0.454) |
| ≥70 years | 0.845 (0.804, 0.889) | 1.000 (Ref.) | 0.860 (0.714, 1.036) | 0.462 (0.349, 0.612) |
| P_interation_ | <0.001 | | | |

Abbreviations: CVH, cardiovascular health; T2D, type 2 diabetes; HR, hazard ratio; 95%CI, 95% confidence interval.

Model 1 was adjusted for age and sex; Model 2 was adjusted for age, sex, living region, and urban area; Model 3 was adjusted for age, sex, living region, urban area, education level, income level, alcohol drinking status, family history of T2D (not included in the model stratified by family history), and subcohorts.

*P*_interaction_ represented the significance of interaction effect between CVH and age groups.

Figure S1 The research concept and process

Abbreviations: China-PAR, The Prediction for Atherosclerotic Cardiovascular Disease Risk in China.

Blood glucose was not included in the calculation of CVH score when exploring the association between family history and the age onset of type 2 diabetes.

Figure S2 Flow diagram of the study population for the analyses

Abbreviations: CVD, cardiovascular disease.

Figure S3 The differences in the onset age of T2D among population with different family history status and CVH level

Abbreviations: RMST, restricted mean survival time; 95%CI, 95% confidence interval.

Figure S4 The association of T2D with different onset age with CVD risk among males (A) and females (B)

Abbreviations: T2D, type 2 diabetes; CVD, cardiovascular disease; HR, hazard ratio; 95%CI, 95% confidence interval; BMI, body mass index.

HRs (95% CIs) for CVD risk associated with T2D across three age groups were calculated using Cox regression models, with population without T2D serving as the reference group. Models were adjusted for urban area, education level, current smoking status, alcohol drinking status, physical activity, family history of CVD, BMI, hypertension, dyslipidemia, and hypoglycemic medication use.

*P*_interaction_ represented the significance of interaction effect between onset age and T2D.

Figure S5 The association of CVH components with CVD risk among T2D patients with different onset age

Abbreviations: CVH, cardiovascular health; CVD, cardiovascular disease; T2D, type 2 diabetes; HR, hazard ratio; 95%CI, 95% confidence interval.

Models were adjusted for age, sex, living region, urban area, education level, alcohol drinking status, family history of CVD and subcohorts.

*P*_interaction_ represented the significance of interaction effect between CVH components and age groups.

Figure S6 The association of CVH score with CVD risk among (A) male and (B) female patients with T2D with different onset age

Abbreviations: CVH, cardiovascular health; CVD, cardiovascular disease; T2D, type 2 diabetes; HR, hazard ratio; 95%CI, 95% confidence interval.

HRs (95% CIs) for CVD risk associated with high CVH across three age groups were calculated using Cox regression models, with population with low CVH serving as the reference group. Models were adjusted for age, living region, urban area, education level, alcohol drinking status, family history of CVD and subcohorts.

*P*_interaction_ represented the significance of interaction effect between CVH and age groups.

Figure S7 The association of T2D with different onset age with CVD risk among total population (A), individuals with (B) and without (C) family history using competing risk model

Abbreviations: CVD, cardiovascular disease; T2D, type 2 diabetes; HR, hazard ratio; 95%CI, 95% confidence interval.

HRs (95% CIs) for CVD risk associated with T2D across three age groups were calculated using competing risk models, with population without T2D serving as the reference group. Models were adjusted for urban area, education level, current smoking status, alcohol drinking status, physical activity, family history of CVD, BMI, hypertension, dyslipidemia, and hypoglycemic medication use.

*P*_interaction_ represented the significance of interaction effect between onset age and T2D.

Figure S8 The association of CVH score with CVD risk among T2D patients with different onset age using competing risk model

Abbreviations: CVH, cardiovascular health; CVD, cardiovascular disease; T2D, type 2 diabetes; HR, hazard ratio; 95%CI, 95% confidence interval.

HRs (95% CIs) for CVD risk associated with high CVH across three age groups were calculated using competing risk models, with population with low CVH serving as the reference group. Models were adjusted for age, sex, living region, urban area, education level, alcohol drinking status, family history of CVD and subcohorts.

The reference group was population with low CVH.

*P*_interaction_ represented the significance of interaction effect between CVH and age groups.
